# Echocardiographic Assessment of Cardiac Structure and Function of Centenarians: A Systematic Review

**DOI:** 10.3390/geriatrics10010026

**Published:** 2025-02-12

**Authors:** Andrea Sonaglioni, Gian Luigi Nicolosi, Giovanna Elsa Ute Muti-Schünemann, Alessio Polymeropoulos, Michele Lombardo, Paola Muti

**Affiliations:** 1 Division of Cardiology, IRCCS MultiMedica, 20123 Milan, Italy; michele.lombardo@multimedica.it; 2 Division of Cardiology, Policlinico San Giorgio, 33170 Pordenone, Italy; gianluigi.nicolosi@gmail.com; 3 Department of Emergency, Fondazione IRCSS Ca’ Granda, Ospedale Maggiore Policlinico, 20122 Milan, Italy; giovanna.muti@unimi.it; 4 Fondazione IRCCS Istituto Nazionale dei Tumori, 20133 Milan, Italy; alessio.polymeropoulos@istitutotumori.mi.it; 5 Department of Biomedical, Surgical and Dental Sciences, University of Milan, 20122 Milan, Italy; pmuti26@gmail.com; 6 IRCCS MultiMedica, 20138 Milan, Italy

**Keywords:** centenarians, echocardiography, cardiac remodeling, systolic function, HFpEF

## Abstract

**Background:** During the last two decades, a limited number of studies have provided echocardiographic details regarding the cardiac structure and function of individuals aged ≥100 years. These studies analyzed limited sample sizes of centenarians using different methodologies. The present systematic review was primarily designed to summarize the main findings of these studies and to examine the overall influence of extremely advanced age on cardiac structure and function. **Methods:** All echocardiographic studies that evaluated the cardiac structure and function in individuals aged ≥100 years, selected from the PubMed, Embase, Scopus and Cochrane Central Register of Controlled Trials (CENTRAL) databases, were included. There was no limitation on the time period. The risk of bias was assessed by using the National Institutes of Health (NIH) Quality Assessment Tool for Observational Cohort and Cross-Sectional Studies. **Results:** A total of eight studies with 1340 centenarians [median age 101.4 years (IQR 101–103 years)] met the eligibility criteria and were analyzed. The centenarians were predominantly females [76.3% (IQR 60–85%)] with a small body surface area, long history of hypertension and slightly impaired renal functional reserve. The centenarian population showed a reduced burden of cardiovascular disease but an increased comorbidity burden, as assessed using the Charlson [median value 3.7 (IQR 1.8–5.5)] and Katz [median value 2.1 (IQR 1.1–3.1)] indexes. The echocardiographic findings comprised left ventricular (LV) concentric remodeling, with first-degree diastolic dysfunction [median E/A ratio 0.8 (IQR 0.7–0.9)], a moderate increase in LV filling pressure [median E/e’ ratio 16.8 (IQR 16.2–17)], normal LV systolic function [median left ventricular ejection fraction (LVEF) 60.9% (IQR 55–84%)] and mild-to-moderate pulmonary hypertension [median systolic pulmonary artery pressure 42.1 mmHg (IQR 37–54 mmHg)]. The pooled prevalence of LV systolic dysfunction (LVEF < 50%) was 15.8%. Moderate-to-severe valvular heart diseases were detected in less than one-third of the centenarians. Compared with the outpatient and in-home cohorts, hospitalized centenarians were less commonly females and were more likely to be affected by significant LV hypertrophy with a supra-normal LVEF, higher degrees of valvulopathies and impaired pulmonary hemodynamics. **Conclusions:** The evidence currently suggests that centenarians have typical LV concentric remodeling with increased myocardial stiffness and diastolic dysfunction, which predispose them to heart failure with a preserved ejection fraction (HFpEF). Cardioprotective treatment should be considered for personalized implementation and uptitration in this special population.

## 1. Introduction

The mean age of the current global population is rapidly increasing. According to the World Health Organization (WHO), the number of people aged 80 years and older is expected to triple between 2020 and 2050, reaching 426 million [1]. More remarkably, the number of centenarians has doubled every decade since the 1950s and is estimated to increase five-fold by the year 2050 [2]. Due to the increase in prevalence of centenarians, they are regularly encountered in clinical practice [3]. Studies on longevity demonstrated that the interaction between genetic and environmental factors favors aging [4,5]. Among this particular population, cardiovascular disease is frequent and represents the most common cause of mortality [6,7,8]. Transthoracic echocardiography (TTE) is an essential tool for the evaluation of age-associated structural and functional cardiac alterations. This noninvasive imaging modality allows for obtaining useful information on cardiac chamber internal dimensions, left ventricular (LV) diastolic function, biventricular systolic function, morphology and function of the heart valves, and pulmonary hemodynamics. To date, cardiac remodeling has been poorly investigated in the “oldest-old” individuals [9]. During the last two decades, a few echocardiographic studies provided detailed assessments on the cardiac structure and function of individuals aged ≥100 years. These studies comprised limited sample sizes and used different methodologies. The present systematic review was primarily designed to summarize the main findings of these studies and examine the overall influence of extremely advanced age on cardiac structure and function. The pathophysiological mechanisms underpinning the cardiac remodeling that occurs in centenarians are discussed as well.

## 2. Methods

This systematic review was performed in accordance with the recommendations of the PRISMA guidelines [10] and was registered in the INPLASY database on 3 November 2024 (registration number INPLASY2024110010).

### 2.1. Search Strategy

A comprehensive search of all echocardiographic studies that evaluated the cardiac structure and function of individuals aged ≥100 years was carried out by two independent reviewers (A.S. and M.L.) up to November 2024 by using the PubMed, Embase, Scopus and Cochrane Central Register of Controlled Trials (CENTRAL) databases. The search was last updated on 11 November 2024. The search strategy included the following terms: “centenarians” AND “transthoracic echocardiography” AND “cardiac function” OR “left ventricular ejection fraction”. Published studies were sought after with the language restriction to English. Forward and backward citation searches were conducted. 

### 2.2. Eligibility Criteria

Studies were included for review if they met the following criteria: they evaluated the echocardiographic characteristics of centenarians, regardless of the time frame. Studies were excluded based on predefined criteria: non-English language, if they were focused on centenarians without or with incomplete echocardiographic data, studies that involved individuals aged <100 years, non-clinical articles, animal studies, duplicate articles, systematic and narrative reviews, case reports, conference presentations, correspondences, editorials, letters without data and abstracts.

### 2.3. Study Selection and Data Extraction

Two reviewers (A.S. and M.L.) independently performed title and abstract and full text screenings of the aforementioned databases according to the inclusion criteria. Data extraction was independently performed for the following information concerning centenarians: (1) demographics (age and sex); (2) anthropometrics [body surface area (BSA) and body mass index (BMI)]; (3) prevalence of the most common cardiovascular risk factors (hypertension, smoking, type 2 diabetes and dyslipidemia); (4) previous history of coronary artery disease (CAD), heart failure and/or transient ischemic attack (TIA)/stroke; (5) comorbidity burden and comorbidity indexes, such as the Charlson comorbidity index (CCI) [11] and the Katz index [12]; (6) physical examination [systolic blood pressure (SBP), diastolic blood pressure (DBP) and heart rate]; (7) electrocardiographic (ECG) data, such as the cardiac rhythm and the pattern of intraventricular conduction; (8) biochemical parameters, such as the serum levels of hemoglobin, creatinine, cholesterol and N-terminal pro-B-type natriuretic peptide (NT-proBNP) or brain natriuretic peptide (BNP); (9) TTE parameters, including the cardiac chambers’ cavity sizes, LV diastolic function and left ventricular filling pressures (LVFPs) (measured by the E/A ratio and E/e’ ratio, respectively), biventricular systolic function assessed by left ventricular ejection fraction (LVEF) and tricuspid annular plane systolic excursion (TAPSE), degree of concomitant valvular heart disease and pulmonary hemodynamics; (10) the current medical treatment; and (11) follow-up data (if any). Possible discrepancies between reviewers were resolved through a consensus discussion with the involvement of a third author (G.L.N.), who checked the extracted data to ensure accuracy, completeness and consistency. 

### 2.4. Quality Assessment

Articles included in this systematic review were assessed for the risk of bias using the National Institutes of Health (NIH) Quality Assessment Tool for Observational Cohort and Cross-Sectional Studies [13]. The quality rating was independently estimated by two authors (A.S. and G.L.N.). Cohen’s Kappa coefficient was used to measure the level of agreement between the two raters and was calculated using k = (p_o_ − p_e_)/(1 − p_e_), where p_o_ is the relative observed agreement between the raters and p_e_ is the probability of random agreement. Moreover, we used the GRADE methodology to assess the certainty of the body of evidence by outcome and produce an evidence profile and interactive summary of the findings [14].

**Figure 1:**
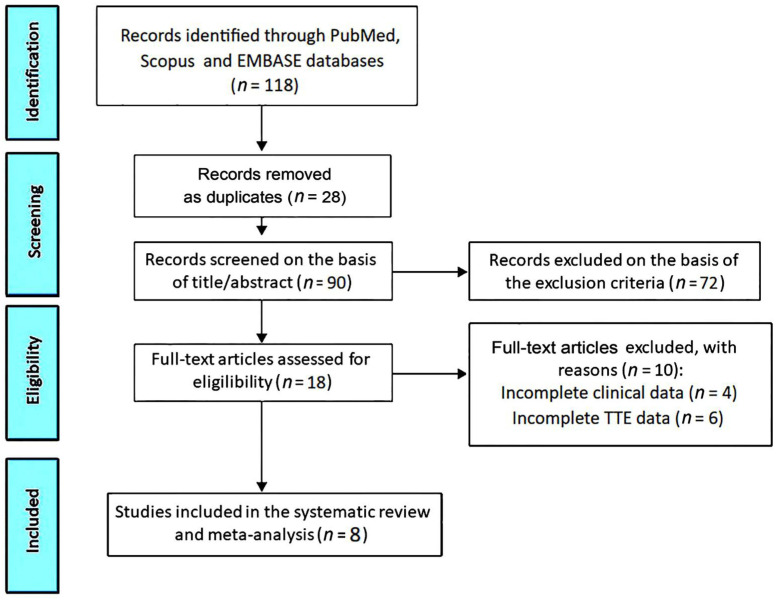
Flow diagram used for identifying the included studies. TTE, transthoracic echocardiography.

The PRISMA flow diagram used for identifying the included studies is depicted in Figure 1.

### 2.5. Statistical Analysis

For continuous variables, summary statistics were calculated using the median (range [min–max]) or mean (SD), whereas for categorical variables, frequency counts (N) or percentages (%) were used as appropriate. Two subgroups of studies that assessed the clinical and echocardiographic data of inpatients vs. outpatients/in-home centenarians were summarized separately using standard descriptive statistics. In addition, in order to account for the heterogeneity of these two subgroups, we performed a two-step subgroup meta-analysis of the outcomes of interest. Specifically, the subgroup analysis was performed using a mixed-effects model (random-effects model within subgroups, fixed-effects model between subgroups). First, the standard errors (SEs) of the effect estimates were calculated using standard formulas for means or proportions. We then pooled effect estimates with their SEs for the inpatients and outpatients/in-home cohorts, and then compared using a z-test. Due to the incomplete data for several clinical and echocardiographic parameters, the comparison between the two subgroups of centenarians was performed for those continuous or categorical parameters that were collected by at least two studies for each sub-group. Two-sided *p*-values below 0.05 were considered statistically significant. The statistical analysis was performed using Comprehensive Meta-Analysis version 3.0 (Biostat, Englewood, NJ, USA), and the meta-analysis was performed using the R programming language, version 4.3.2 (www.r-project.org/), using the function rma of the (“metafor”) package version 4.6-0.

## 3. Results

### 3.1. Clinical Findings

The database searches were run from inception to 11 November 2024 and identified 118 references. The title and abstract phase, performed in duplicate by the screeners A.S. and M.L., saw the exclusion of 100 studies. At the full text stage, the number of studies assessed for eligibility was 18 (15.2%), of which 10 (8.5%) were excluded. The most common reasons for exclusion were the following: studies conducted on centenarians without echocardiographic data (61%), duplicates (15.2%), incomplete clinical data (3.4%) and incomplete TTE data (5.1%). A total of eight studies (6.8%) [15,16,17,18,19,20,21,22] were thus included in this systematic review, totaling 1340 centenarians. The included populations were composed of 37% (3/8) retrospective cohort studies [15,17,21] and 63% (5/8) prospective cohort studies [16,18,19,20,22]. Table 1 summarizes the main findings of the eight studies included in the present systematic review. 

The included studies were published between 2007 and 2023. Three studies were performed in the USA; two in Italy; and one each in Spain, Denmark and China. The median age of the centenarians at the echocardiographic assessment was 101.4 years (IQR 100.1–103 years). Females comprised 76.3% of the centenarians (IQR 60–85%). Three studies [19,20,22] analyzed in-home centenarians, two studies [16,17] outpatients and two studies [15,18] inpatients, whereas the remaining study of Perez J et al. [21] analyzed an equal proportion of outpatients and inpatients. Three studies [18,19,22] performed echocardiography by using a Philips CX-50 portable echocardiograph, whereas Perez J et al. [21] used a General Electric (GE) cardiovascular ultrasound system; the remaining studies did not specify the ultrasound system employed for the echocardiographic evaluation of centenarians. Among the included studies, only two authors [16,17] provided follow-up data, whereas the great majority of studies (75% of the total) were primarily focused on the echocardiographic assessment of centenarian hearts at the baseline.

The baseline demographic, anthropometric, clinical and biochemical parameters concerning centenarians collected by the included studies are reported in Table 2.

Overall, the centenarians were predominantly females with a small BSA; with a moderate-to-high prevalence of hypertension; and with low prevalences of smoking, type 2 diabetes and dyslipidemia. The analysis of comorbidities revealed a high comorbidity burden [median CCI 3.7 (IQR 1.8–5.5)] and severe functional impairment [median Katz index 2.1 (IQR 1.1–3.1)]. The physical examinations showed normal median values of both the SBP and DBP and a normal median heart rate. The pooled prevalence of atrial fibrillation (AF) was 26.7%. Information concerning the biochemical parameters and current medical treatment were only provided in a limited number of studies, ranging from 12.5% to 36.5% of the total. Blood test results demonstrated a slightly impaired renal functional reserve, optimal lipid panel and increased serum levels of NT-proBNP/BNP. With regard to the current medical treatment, we commonly found angiotensin-converting enzyme inhibitors (ACEis) or angiotensin receptor blockers (ARBs), followed by diuretics and beta blockers, whereas antiplatelets, anticoagulants, digoxin and statins were administered in less than one-third of the centenarians.

### 3.2. Transthoracic Echocardiography Findings

Data regarding LV thickness/internal dimensions and LV systolic function were provided by a percentage of studies that ranged from 87.5% to 100% of the total, whereas the remaining echocardiographic parameters were assessed by a number of studies that ranged from 25% to 75% of the total. 

Table 3 lists all the conventional echo Doppler parameters obtained by the included studies on the centenarians.

The TTE examination revealed moderate LV hypertrophy with concentric remodeling, mild left atrial (LA) dilatation and a normal right chamber cavity size. The impaired relaxation pattern of the transmitral flow [median E/A ratio 0.8 (IQR 0.7–0.9)] was the most common LV filling pattern detected in centenarians, whereas the LVFPs were moderately increased, as expressed by the E/e’ ratio [median value 16.8 (IQR 16.2–17)]. Seven studies (87.5%) measured the LVEF using the Simpson biplane method, while only Sadiq A et al. [15] used the Teichholz method for calculating the LVEF. Overall, the LVEF [median value 60.9% (IQR 55–84%)] was preserved, while LV systolic dysfunction, defined as LVEF < 50% [23], was detected in 15.8% of the centenarians. Moderate-to-severe aortic stenosis, mitral regurgitation and tricuspid regurgitation were detected in approximately one out of five centenarians. An analysis of the pulmonary hemodynamics showed mild-to-moderate pulmonary hypertension [median systolic pulmonary artery pressure (sPAP) 42.1 mmHg (IQR 37–54 mmHg)]. Finally, the aortic root diameter was slightly increased in comparison with the accepted reference values for healthy individuals aged ≥80 years [24].

In the MILANO study [18], our study group demonstrated a reduced prevalence of echocardiographic congestive signs among hospitalized centenarians. Interestingly, the centenarians were frequently diagnosed with an increased systolic midventricular pressure gradient, likely related to a chronically reduced preload due to chronic dehydration and/or hypovolemia with consequent supra-normal ventricular emptying. Moreover, we measured the arterial elastance index (EaI), LV end-systolic elastance index (EesI) and resultant EaI/EesI ratio (V-A coupling) in the centenarian cohort and compared the results with a group of sex-matched hospitalized octogenarians. The VAC parameters were significantly increased in centenarians vs. octogenarians.

### 3.3. Subgroup Analysis

Pooled estimates of the principal clinical and echocardiographic variables obtained by the included studies in the two cohorts of inpatients and outpatients/in-home centenarians are reported in Table 4. 

The subgroup analysis suggested a slightly lower proportion of females among the hospitalized centenarians compared with the outpatients and in-home centenarians, although this difference was not statistically significant. Moreover, the inpatients showed a higher prevalence of smoking history. The burden of cardiovascular and noncardiovascular comorbidities was similar in the subgroups of centenarians. 

Using TTE, the inpatients were diagnosed with a greater degree of LV hypertrophy and a significantly higher E/A ratio and sPAP magnitude compared with the outpatient/in-home cohorts. The LVEF tended to be higher in the hospitalized centenarians than in the non-hospitalized cohorts, even if this increase was not statistically different. Finally, moderate-to-severe aortic regurgitation, mitral regurgitation and tricuspid regurgitation were more frequently detected in the hospitalized centenarians. 

### 3.4. Follow-Up Data

Concerning the follow-up data, Martínez-Sellés M et al. [16] found that roughly half of the population of centenarians had died during the first year of follow-up. Cox regression analysis revealed that age, the Charlson and Katz indexes, systolic dysfunction and severe aortic regurgitation were independent predictors of mortality. The study by Brenes-Salazar JA et al. [17] demonstrated 1-month and 1-year mortalities of 15% and 47%, respectively; the median survival after the echocardiogram was 13 months, independent of management. Accordingly, the authors concluded that echocardiography influenced the medical decision making and management only in a minority of cases, with a minimal impact on the overall care of the centenarians.

### 3.5. Risk of Bias Assessment

The quality of the included studies was judged to have a low risk of bias for the study of Martínez-Sellés M et al. [16] and fair for the remaining seven studies. The Cohen’s Kappa coefficient for the agreement between the reviewers in the risk of bias assessment was interpreted as a substantial agreement, with k = 0.80.

### 3.6. GRADE Assessment

The body of evidence regarding the echocardiographic features of centenarians was estimated to be of moderate certainty of the evidence (Table 5).

**Question**: echocardiographic studies performed in cohorts of hospitalized and non-hospitalized centenarians for the detection of age-related cardiac remodeling.
**Setting:** inpatients, outpatients and in-home centenarians.

## 4. Discussion

### 4.1. Main Findings of This Systematic Review

The present systematic review included eight echocardiographic studies conducted on individuals aged ≥100 years over a 16-year period. Our results reveal that the centenarians were mostly females with a small BSA, a long history of hypertension and a slightly impaired renal functional reserve. These had a reduced cardiovascular disease burden with an optimal blood pressure control and lipid profile, but an increased comorbidity burden. The TTE demonstrated LV hypertrophy with first-degree diastolic dysfunction, a moderate increase in LVFPs, normal LV systolic function and mild-to-moderate pulmonary hypertension. LV systolic dysfunction and moderate-to-severe valvular heart diseases were rarely detected. Additionally, our study group [18] demonstrated an increased prevalence of systolic midventricular pressure gradients associated with increased arterial and end-systolic stiffness in hospitalized centenarians.

Compared with the outpatient and in-home cohorts, the hospitalized centenarians were less commonly females and were more likely to be affected by significant LV hypertrophy with supra-normal LVEFs, higher degrees of valvulopathies and impaired pulmonary hemodynamics.

Based on the findings of the two studies that provided follow-up data [16,17], the centenarians had a mortality rate of about 50% over the 1-year follow-up. Advanced age, increased comorbidity burden, LV systolic dysfunction and moderate-to-severe valvular heart diseases were independently associated with mortality in the centenarians. 

The included studies demonstrated the high feasibility of TTE, which allowed for a comprehensive evaluation of the centenarians’ cardiac chambers’ sizes, systolic and diastolic functions, valvular performance and hemodynamics. Both the hospitalized and non-hospitalized cohorts of centenarians were diagnosed with a common echocardiographic phenotype of LV hypertrophy, that is, the LV concentric remodeling.

### 4.2. Pathophysiological Mechanisms Underpinning LV Concentric Remodeling in Centenarians

**Figure 2:**
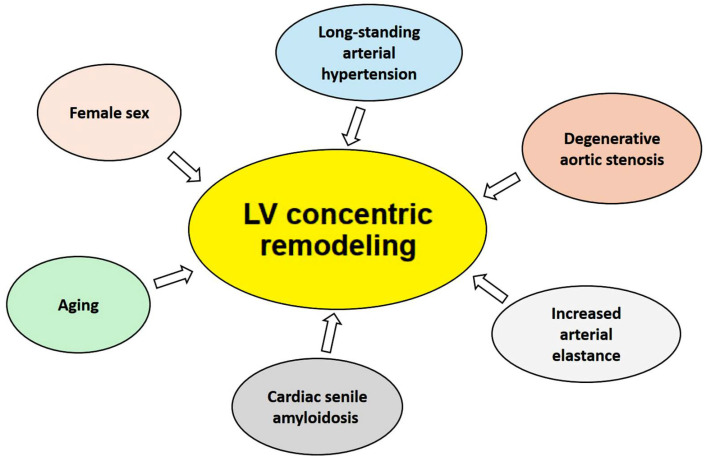
Pathophysiological mechanisms underpinning LV concentric remodeling in centenarians.

The LV concentric remodeling detected in centenarians by echocardiography may be considered as the result of several pathophysiological mechanisms (Figure 2). 

First, the aging process is itself associated with an increase in RWT and LV concentric remodeling, characterized by progressive myocyte dropout, myocyte hypertrophy and increased collagen concentration, as demonstrated by histomorphometric studies in animals and humans [25,26,27]. Second, female sex is associated with LV concentric remodeling in the general population, irrespective of the burden of cardiovascular risk factors [28,29]. The LV concentric remodeling observed in centenarians may also represent a cardiac adaptation to long-standing arterial hypertension [30] and/or degenerative aortic stenosis [31]. An increased arterial elastance secondary to degeneration and hyperplasia of the arterial wall may also be another cause of increased LV afterload, leading to LV hypertrophy [32,33]. LV concentric remodeling in elderly individuals may also be the phenotypic expression of cardiac senile amyloidosis, an infiltrative cardiomyopathy characterized by extracellular amyloid infiltration throughout the heart [34]. LV hypertrophy in centenarians is associated with a significant increase in myocardial stiffness and abnormalities in diastolic function, including impaired LV relaxation, increased LVFPs, LA remodeling and postcapillary pulmonary hypertension [35,36]. The combined increase in ventricular and arterial stiffening promotes load-induced impairment in LV relaxation, with a consequent increase in the serum levels of NT-proBNP/BNP [37,38]. 

An analysis of the LV systolic function by TTE showed that the pooled LVEF was preserved in centenarians. However, the subgroup analysis revealed that compared with the outpatient and in-home cohorts, the hospitalized cohorts were more likely to be diagnosed with a supra-normal LVEF (≥65%). The supra-normal LV contractile phenotype is a newly recognized state associated with an unfavorable prognosis, as reported by recent studies conducted on both chronic [39,40] and acute [41,42] heart failure patients. These studies highlighted significant pathophysiological differences between heart failure patients with LVEF 50% to 64% (the “normal” LVEF phenotype) in comparison with those with EF ≥ 65% (the “supra-normal” LVEF phenotype). However, these studies evaluated the prognostic role of a “supra-normal” LVEF, predominantly in middle-aged participants (aged <70 years), without including the “oldest-old” individuals. The results of the present systematic review would confirm that the “normal” LVEF phenotype, commonly detected in non-hospitalized centenarians, is an important clinical predictor of long-term survival. On the other hand, the “supra-normal” LVEF phenotype has been described in hospitalized centenarians with infectious disease related to pulmonary or non-pulmonary infections. The infective process associated with elevated body temperatures and fluid loss is responsible for dehydration, hypovolemia and a reduction in preload, thus causing a hypercontractile response [18]. In this clinical context, TTE generally shows severe LV hypertrophy, a small LV cavity size, severe diastolic dysfunction and a dynamic intraventricular obstruction. The “supra-normal” LVEF phenotype was also recently described in heart failure patients with the “cold-dry” hemodynamic phenotype, a negative prognostic indicator over the mid-term follow-up period [43].

### 4.3. Implications for Clinical Practice

The progressive increase in both arterial elastance and myocardial stiffness detected in centenarians may result in a decreased exercise tolerance and enhanced load sensitivity [44,45]. Indeed, in patients with stiff noncompliant ventricles, sudden changes in LV afterload or LV preload may cause uncontrolled increases in LVFPs, resulting in pulmonary edema [46]. For these reasons, centenarians are particularly susceptible to heart failure with apreserved ejection fraction (HFpEF) [47,48]. In this regard, TTE may help the clinicians to identify those who are at an increased risk of cardiovascular complications among the oldest-old patients. Additionally, as indicated by the literature data [49,50,51], a comprehensive echocardiographic assessment of cardiac chambers’ cavity sizes, systolic and diastolic function, valvular function and pulmonary hemodynamics might allow the clinicians to select a limited number of elderly individuals who could benefit from interventional procedures.

TTE may also guide the clinicians in selecting and/or uptitrating the most appropriate medical treatment for centenarians. In this regard, the echocardiographic findings of small and stiff cardiac chambers with an intraventricular pressure gradient might suggest the clinicians use beta blockers and ensure adequate volemic filling [52]. Moreover, due to the increased risk of fatal bleeding related to decreased renal function, several comorbidities, coexisting frailty and the risk of falls [53,54,55], antiplatelets and anticoagulants should be used with great caution in this special population.

### 4.4. Limitations of the Included Studies 

The main limitations of the included studies were their monocentric nature, the limited sample size of centenarians examined, the retrospective design of 37% of the total and the lack of follow-up data for 75% of the studies. Moreover, the included studies assessed the cardiac structure and function of the centenarians by evaluating the echocardiographic characteristics of heterogeneous study populations, i.e., inpatients, outpatients and in-home centenarians. Most conventional echocardiographic parameters were measured by a limited number of studies, and innovative echocardiographic data were lacking. In particular, no study measured the myocardial strain parameters using speckle-tracking echocardiography (STE) in the centenarians. Due to the higher sensitivity of LV global longitudinal strain (GLS) than LVEF for detecting subclinical LV systolic dysfunction [56], STE analysis could have provided more information regarding the cardiac performance of the centenarians. Even if centenarians exhibit echocardiographic features suggestive of diastolic dysfunction and LV remodeling, which is commonly associated with susceptibility to HFpEF, a definitive diagnosis of HFpEF, including the presence of heart failure symptoms, was not assessed in the included studies. While diastolic dysfunction and LV remodeling indicate a potential predisposition to HFpEF in centenarians, further research incorporating clinical and longitudinal data is necessary to clarify its prevalence and clinical impact.

## 5. Conclusions

Centenarians have typical LV concentric remodeling with an increased myocardial stiffness and diastolic dysfunction, making them particularly susceptible to the development of HFpEF.

TTE may allow clinicians to obtain a comprehensive evaluation of both hospitalized and non-hospitalized centenarians by providing a detailed assessment of the cardiac chambers’ sizes, systolic and diastolic functions, valvular performance and hemodynamics. 

Medical treatment should be considered for personalized implementation and uptitration in this special population.

## Figures and Tables

**Table 1:** Main findings of the studies included in the present systematic review. EaI, arterial elastance index; EesI, end-systolic elastance index; IVST, interventricular septum thickness; LA, left atrial; LV, left ventricular; LVEF, left ventricular ejection fraction; LVFP, left ventricular filling pressure; RWT, relative wall thickness; VAC, ventricular–arterial coupling; VHD, valvular heart disease.

Study Name and Country	Median Age (Years)	Size (% Females)	Study Design	Study Population	Echocardiographic Features of Centenarians
Sadiq A et al. (2007) [15], USA	101	63 (60)	Retrospective	100% inpatients	Small LV size, supernormal LVEF, pulmonary hypertension, higher prevalence of VHD in men
Martínez-Sellés M et al. (2015) [16], Spain	101.5	118 (76.3)	Prospective	100% outpatients	LV hypertrophy, LV diastolic dysfunction, normal LVEF, LA dilatation, pulmonary hypertension
Brenes-Salazar JA et al. (2018) [17], USA	101	114 (72)	Retrospective	100% outpatients	Normal LVEF, bi-atrial enlargement, pulmonary hypertension and valve abnormalities
Sonaglioni et al. (2018) [18], Italy	100.5	118 (80)	Prospective	100% inpatients	High degree of RWT, normal LVEF, high EaI, EesI and VAC, pulmonary hypertension
Rasmussen SH et al. (2019) [19], Denmark	100.1	125 (78)	Prospective	100% in-home centenarians	LV hypertrophy, LV diastolic dysfunction, normal LVEF, LA dilatation, pulmonary hypertension
Cannatà A et al. (2020) [20], Italy	103	20 (85)	Prospective	100% in-home centenarians	Diastolic dysfunction, normal LVEF, heart failure in one-third of centenarians
Perez J et al. (2022) [21], USA	101.5	100 (78)	Retrospective	50% inpatients 50% outpatients	Preserved LV size and systolic function, elevation in LVFP mild pulmonary hypertension
Sun Z et al. (2023) [22], China	102.3	682 (81.2)	Prospective	100% in-home centenarians	Depressive disorder in 26.2% of centenarians, higher LVEF and IVST in centenarians with depression

**Table 2:** Baseline demographic, anthropometric, clinical and biochemical parameters concerning centenarians collected by the included studies. Categorical variables are reported as the pooled prevalence (%), while continuous data are expressed as the median and interquartile range. ACEis, angiotensin-converting-enzyme inhibitors; AF, atrial fibrillation; ARBs, angiotensin receptor blockers; BB, beta blockers; BMI, body mass index; BNP, brain natriuretic peptide; BSA, body surface area; CAD, coronary artery disease; CCI, Charlson comorbidity index; COPD, chronic obstructive pulmonary disease; DBP, diastolic blood pressure; HDL, high-density lipoprotein; LBBB, left bundle branch block; LDL, low-density lipoprotein; NT-proBNP, N-terminal pro-B-type natriuretic peptide; SBP, systolic blood pressure; TIA, transient ischemic attack.

	Centenarians	Number of Studies for Parameters Assessed (%)
Age (years)	101.4 (101–103)	8 (100)
Female sex (%)	76.3	8 (100)
BSA (m^2^)	1.60 (1.56–1.65)	3 (37.5)
BMI (kg/m^2^)	20.8 (14–24.8)	3 (37.5)
Hypertension (%)	64.7	6 (75)
Previous smoking (%)	9.7	4 (50)
Type 2 diabetes (%)	10.3	7 (87.5)
Dyslipidemia (%)	18.1	2 (25)
History of CAD (%)	22.4	7 (87.5)
Previous TIA/stroke (%)	15.8	5 (62.5)
COPD (%)	21.1	3 (37.5)
Cognitive impairment/dementia (%)	55.3	3 (37.5)
Physical disability (%)	48.5	3 (37.5)
CCI	3.7 (1.8–5.5)	2 (25)
Katz index	2.1 (1.1–3.1)	2 (25)
SBP (mmHg)	127 (118–130.6)	5 (62.5)
DBP (mmHg)	70.2 (63–78.9)	5 (62.5)
Heart rate (bpm)	76.2 (71–80)	3 (37.5)
AF (%)	26.7	6 (75)
LBBB (%)	9.9	2 (25)
Pacemaker rhythm (%)	3.8	2 (25)
Hemoglobin (g/dL)	11.5 (11.2–11.9)	3 (37.5)
Creatinine (mg/dL)	1.2 (1.05–1.3)	3 (37.5)
Total cholesterol (mg/dL)	160 (157–163)	2 (25)
LDL-cholesterol (mg/dL)	87 (80–94)	2 (25)
HDL-cholesterol (mg/dL)	47.8 (47.6–48)	2 (25)
NT-proBNP (pg/mL)	3911 (1990–5832)	2 (25)
BNP (pg/mL)	146 (75–217)	2 (25)
Antiplatelets (%)	28.2	3 (37.5)
Anticoagulants (%)	17.8	1 (12.5)
ACEis/ARBs (%)	40.7	3 (37.5)
BB (%)	29	2 (25)
Digoxin (%)	11	2 (25)
Statins (%)	13.4	2 (25)
Diuretics (%)	39	3 (37.5)

**Table 3:** Conventional echo Doppler parameters measured in the centenarians by the included studies. Categorical variables are reported as the pooled prevalence (%), while continuous data are expressed as the median and interquartile range. A-P, antero-posterior; AR, aortic regurgitation; AS, aortic stenosis; IVS, interventricular septum; LA, left atrial; LAVi, left atrial volume index; LVEDD, left ventricular end-diastolic diameter; LVEF, left ventricular ejection fraction; LVESD, left ventricular end-systolic diameter; LVMi, left ventricular mass index; MR, mitral regurgitation; MS, mitral stenosis; PW, posterior wall; RVEDD, right ventricular end-diastolic diameter; RWT, relative wall thickness; sPAP, systolic pulmonary artery pressure; TAPSE, tricuspid annular plane systolic excursion; TR, tricuspid regurgitation.

Transthoracic Echocardiography	Centenarians	Number of Studies for Parameters Assessed (%)
IVS thickness (mm)	12.3 (9.6–16)	7 (87.5)
PW thickness (mm)	10.5 (9–12.1)	7 (87.5)
LVEDD (mm)	42.7 (37.8–56)	7 (87.5)
LVESD (mm)	27 (18–35)	7 (87.5)
RWT	0.50 (0.36–0.59)	7 (87.5)
LVMi (g/m^2^)	103.9 (87–115)	3 (37.5)
LVEF (%)	60.9 (55–84)	8 (100)
LA A-P diameter (mm)	39.2 (29.8–45)	4 (50)
LAVi (mL/m^2^)	43.1 (34–48.3)	4 (50)
E/A ratio	0.8 (0.7–0.9)	4 (50)
E/e’ ratio	16.5 (16–18)	4 (50)
Moderate-to-severe AS (%)	23	6 (75)
Moderate-to-severe AR (%)	10	6 (75)
Moderate-to-severe MR (%)	19.3	7 (87.5)
Moderate-to-severe MS (%)	2.8	4 (50)
Moderate-to-severe TR (%)	23.3	5 (62.5)
RVEDD (mm)	29.8 (26.8–34)	3 (37.5)
TAPSE (mm)	19.5 (18.1–20.9)	2 (25)
sPAP (mmHg)	42 (32–51)	6 (75)
Aortic root (mm)	33.3 (29.7–35.5)	4 (50)

**Table 4:** The pooled-effect estimates of the main clinical and echocardiographic data collected by the two subgroups of studies evaluating inpatients and outpatients/in-home centenarians. Significant *p*-values are in bold. AF, atrial fibrillation; A-P, antero-posterior; AR, aortic regurgitation; AS, aortic stenosis; CAD, coronary artery disease; IVS, interventricular septum; LA, left atrial; LAVi, left atrial volume index; LVEDD, left ventricular end-diastolic diameter; LVEF, left ventricular ejection fraction; LVESD, left ventricular end-systolic diameter; MR, mitral regurgitation; MS, mitral stenosis; PW, posterior wall; RWT, relative wall thickness; SBP, systolic blood pressure; SE, standard error; sPAP, systolic pulmonary artery pressure; TIA, transient ischemic attack; TR, tricuspid regurgitation.

	Inpatients	Outpatients/In-Home Centenarians	*p*-Value
**Age (years)**			
Mean (SE)	100.9 (0.522)	101.5 (0.412)	0.447
**Females**			
Prevalence (SE)	0.74 (0.036)	0.78 (0.025)	0.432
**Hypertension**			
Prevalence (SE)	0.66 (0.057)	0.64 (0.044)	0.796
**Diabetes**			
Prevalence (SE)	0.10 (0.035)	0.09 (0.027)	0.913
**Previous smoking**			
Prevalence (SE)	0.13 (0.032)	0.04 (0.025)	**0.025**
**AF**			
Prevalence (SE)	0.26 (0.051)	0.27 (0.039)	0.855
**CAD history**			
Prevalence (SE)	0.31 (0.097)	0.16 (0.073)	0.210
**Previous TIA/stroke**			
Prevalence (SE)	0.13 (0.057)	0.16 (0.046)	0.639
**SBP (mmHg)**			
Mean (SE)	128.9 (2.77)	125.5 (3.69)	0.459
**IVS thickness (mm)**			
Mean (SE)	12.7 (1.28)	11.8 (1.11)	0.623
**PW thickness (mm)**			
Mean (SE)	11.3 (0.468)	9.78 (0.410)	**0.013**
**LVEDD (mm)**			
Mean (SE)	40.5 (3.53)	43.9 (3.08)	0.468
**LVESD (mm)**			
Mean (SE)	22.3 (3.67)	29.9 (3.01)	0.110
**RWT**			
Mean (SE)	0.56 (0.039)	0.45 (0.033)	**0.038**
**LVEF (%)**			
Mean (SE)	65.9 (5.32)	57.8 (4.11)	0.229
**LA A-P diameter (mm)**			
Mean (SE)	43 (4.24)	35.3 (4.20)	0.198
**LAVi (m** **L** **/m^2^)**			
Mean (SE)	41.1 (5.45)	44.9 (5.34)	0.619
**E/A ratio**			
Mean (SE)	0.85 (0.017)	0.7 (0.017)	**<0.001**
**E/e’ ratio**			
Mean (SE)	16.5 (0.378)	16.8 (0.338)	0.606
**Moderate-to-severe AS**			
Prevalence (SE)	0.25 (0.037)	0.2 (0.033)	0.328
**Moderate-to-severe AR**			
Prevalence (SE)	0.14 (0.035)	0.06 (0.029)	**0.037**
**Moderate-to-severe MR**			
Prevalence (SE)	0.33 (0.054)	0.08 (0.044)	**<0.001**
**Moderate-to-severe MS**			
Prevalence (SE)	0.039 (0.026)	0.006 (0.026)	0.385
**Moderate-to-severe TR**			
Prevalence (SE)	0.32 (0.040)	0.10 (0.040)	**<0.001**
**sPAP (mmHg)**			
Mean (SE)	47.8 (1.84)	36.6 (1.88)	**<0.001**
**Aortic root (mm)**			
Mean (SE)	34.2 (2.09)	32.3 (2.07)	0.513

**Table 5:** GRADE summary of findings based on the included body of evidence. ^a^ A total of 75% of studies performed an echocardiographic evaluation of centenarians at baseline without providing follow-up data. ^b^ Population size was too small to make an inference about the effect of the intervention. ^c^ The retrospective design of many included studies did not permit eliminating the potential confounders that may be present in the results. ^d^ Differences in the modalities of the TTE may also limit the reliability of results since some assessed in-patients with more controlled variables compared with out-patients or in-home patients. Additionally, the ultrasound machines used were not the same for all the included studies, which could have contributed to the differences in measurements. ^e^ Not all studies included all echocardiographic parameters, raising concerns regarding missing data for each participant. ^f^ Major concerns in the risk of bias assessment due to the lack of patient demographic information, such as comorbidities and concomitant medication. HFpEF, heart failure with preserved ejection fraction; LV, left ventricular; TTE, transthoracic echocardiography.

Certainty Assessment							Impact	Certainty	Importance
№ of Studies	Study Design	Risk of Bias	Inconsistency	Indirectness	Imprecision	Other Considerations			
8	Non-randomized studies [15,16,17,18,19,20,21,22]	Fair ^a,b,c,d,e,f^	Not serious	Not serious	Serious ^a,b,d^	All plausible residual confounding would suggest a spurious effect, while no effect was observed.	The body of evidence indicates that the centenarians had typical LV concentric remodeling with increased myocardial stiffness and diastolic dysfunction, making them particularly susceptible to the development of HFpEF. The use of echocardiography may allow clinicians to evaluate the cardiac chambers’ sizes, systolic and diastolic function, valvular performance and hemodynamics, potentially guiding a tailored prescription of cardioprotective treatment. These results, however, require further validation, using a larger population size with additional information regarding comorbidities and concomitant medication [15,16,17,18,19,20,21,22].	⊕⊕⊕○ Moderate ^a,b,c,d,e,f^	Important

## Data Availability

Data extracted from the included studies will be publicly available on Zenodo (https://zenodo.org), pending acceptance by the journal.

## References

[1] Rudnicka E., Napierała P., Podfigurna A., Męczekalski B., Smolarczyk R., Grymowicz M. (2020). The World Health Organization (WHO) approach to healthy ageing. Maturitas.

[2] European Union, Eurostat (2019). Ageing Europe—Looking at the Lives of Older People in the EU.

[3] Rabuñal Rey R., Monte Secades R., Rigueiro Veloso M.T., Casariego Vales E.J., Ibáñez Alonso M.D., García Pais M.J. (2002). Centenarian patients attended at a general hospital. Rev. Clin. Esp..

[4] Cannatà A., Camparini L., Sinagra G., Giacca M., Loffredo F.S. (2016). Pathways for salvage and protection of the heart under stress: Novel routes for cardiac rejuvenation. Cardiovasc. Res..

[5] Cannata A., Merlo M., Artico J., Gentile P., Camparini L., Cristallini J., Porcari A., Loffredo F., Sinagra G. (2018). Cardiovascular aging: The unveiled enigma from bench to bedside. J. Cardiovasc. Med..

[6] Klatt E.C., Meyer P.R. (1987). Geriatric autopsy pathology in centenarians. Arch. Pathol. Lab. Med..

[7] Andersen-Ranberg K., Schroll M., Jeune B. (2001). Healthy centenarians do not exist, but autonomous centenarians do: A population-based study of morbidity among Danish centenarians. J. Am. Geriatr. Soc..

[8] Berzlanovich A.M., Keil W., Waldhoer T., Sim E., Fasching P., Fazeny-Dörner B. (2005). Do centenarians die healthy? An autopsy study. J. Gerontol. A Biol. Sci. Med. Sci..

[9] Fleg J.L., Strait J. (2012). Age-associated changes in cardiovascular structure and function: A fertile milieu for future disease. Heart Fail. Rev..

[10] Moher D., Liberati A., Tetzlaff J., Altman D.G. (2009). Preferred reporting items for systematic reviews and meta-analyses: The PRISMA statement. PLoS Med..

[11] Charlson M.E., Pompei P., Ales K.L., MacKenzie C.R. (1987). A new method of classifying prognostic comorbidity in longitudinal studies: Development and validation. J. Chronic Dis..

[12] Katz S., Ford A.B., Moskowitz R.W., Jackson B.A., Jaffe M.W. (1963). Studies of illness in the aged. the index of adl: A standardized measure of biological and psychosocial function. JAMA.

[13] Ma L.L., Wang Y.Y., Yang Z.H., Huang D., Weng H., Zeng X.T. (2020). Methodological quality (risk of bias) assessment tools for primary and secondary medical studies: What are they and which is better?. Mil. Med. Res..

[14] Guyatt G., Oxman A.D., Akl E.A., Kunz R., Vist G., Brozek J., Norris S., Falck-Ytter Y., Glasziou P., DeBeer H. (2011). GRADE guidelines: 1. Introduction-GRADE evidence profiles and summary of findings tables. J. Clin. Epidemiol..

[15] Sadiq A., Choudhury M., Ali K., Mohamed E., Shetty V., Kabalkin C., Greengart A. (2007). Echocardiographic characteristics in patients > or = 100 years of age. Am. J. Cardiol..

[16] Martínez-Sellés M., García de la Villa B., Cruz-Jentoft A.J., Vidán M.T., Gil P., Cornide L., Ramos Cortés M., González Guerrero J.L., Barros Cerviño S.M., Díaz Castro Ó. (2015). Centenarians and their hearts: A prospective registry with comprehensive geriatric assessment, electrocardiogram, echocardiography, and follow-up. Am. Heart J..

[17] Brenes-Salazar J.A., de la Fuente J., Marella P., Chaliki H., Scott C., Connolly H.M., Click R.L. (2018). Echocardiography in centenarians: Characteristics, utility and follow-up. J. Geriatr. Cardiol..

[18] Sonaglioni A., Lombardo M., Baravelli M., Vincenti A., Rigamonti E., Vanoli E., Nicolosi G.L., Sommese C., Anzà C. (2018). AnatoMy and physIopathoLogy of the heArt in a ceNtenarian cOhort (MILANO study). Am. Heart J..

[19] Rasmussen S.H., Andersen-Ranberg K., Dahl J.S., Nybo M., Jeune B., Christensen K., Gill S. (2019). Diagnosing heart failure in centenarians. J. Geriatr. Cardiol..

[20] Cannatà A., Gentile P., Paldino A., Nuzzi V., Camparini L., Ciucci G., Manca P., Artico J., Dal Ferro M., Marcon G. (2020). Echocardiographic evaluation of centenarians in Trieste. J. Cardiovasc. Med..

[21] Perez J., Hurwitz B., Salguero D., Donattele M., Escolar E., Fernandez R., Mihos C.G. (2022). Echocardiographic Features of Longevity: A Cross-Sectional Study of Centenarians. Cureus.

[22] Sun Z., Ping P., Zhang P., Yao Y., Huang Z., Zhao Y., Luo L., Fu S. (2023). Associations between cardiac structure and function and depressive disorder: A centenarian study in China. Heliyon.

[23] Lang R.M., Badano L.P., Mor-Avi V., Afilalo J., Armstrong A., Ernande L., Flachskampf F.A., Foster E., Goldstein S.A., Kuznetsova T. (2015). Recommendations for cardiac chamber quantification by echocardiography in adults: An update from the American Society of Echocardiography and the European Association of Cardiovascular Imaging. J. Am. Soc. Echocardiogr..

[24] Wu J., Zeng W., Li X., Zhu J., Zhou C., Fan R., Sun T., Fei H., Li X. (2023). Aortic size distribution among normal, hypertension, bicuspid, and Marfan populations. Eur. Heart J. Imaging Methods Pract..

[25] Anversa P., Hiler B., Ricci R., Guideri G., Olivetti G. (1986). Myocyte cell loss and myocyte hypertrophy in the aging rat heart. J. Am. Coll. Cardiol..

[26] Anversa P., Palackal T., Sonnenblick E.H., Olivetti G., Meggs L.G., Capasso J.M. (1990). Myocyte cell loss and myocyte cellular hyperplasia in the hypertrophied aging rat heart. Circ. Res..

[27] Olivetti G., Melissari M., Capasso J.M., Anversa P. (1991). Cardiomyopathy of the aging human heart. Myocyte loss and reactive cellular hypertrophy. Circ. Res..

[28] St Pierre S.R., Peirlinck M., Kuhl E. (2022). Sex Matters: A Comprehensive Comparison of Female and Male Hearts. Front. Physiol..

[29] Martin T.G., Leinwand L.A. (2024). Hearts apart: Sex differences in cardiac remodeling in health and disease. J. Clin. Investig..

[30] Messerli F.H., Aepfelbacher F.C. (1995). Hypertension and left-ventricular hypertrophy. Cardiol. Clin..

[31] Bornstein A.B., Rao S.S., Marwaha K. (2024). Left Ventricular Hypertrophy. StatPearls.

[32] Nichols W.W., Edwards D.G. (2001). Arterial elastance and wave reflection augmentation of systolic blood pressure: Deleterious effects and implications for therapy. J. Cardiovasc. Pharmacol. Ther..

[33] Sonaglioni A., Baravelli M., Lombardo M., Sommese C., Anzà C., Kirk J.A., Padeletti L. (2018). Ventricular-arterial coupling in centenarians without cardiovascular diseases. Aging Clin. Exp. Res..

[34] de Marneffe N., Dulgheru R., Ancion A., Moonen M., Lancellotti P. (2022). Cardiac amyloidosis: A review of the literature. Acta Cardiol..

[35] Borlaug B.A. (2020). Evaluation and management of heart failure with preserved ejection fraction. Nat. Rev. Cardiol..

[36] Lam C.S., Borlaug B.A., Kane G.C., Enders F.T., Rodeheffer R.J., Redfield M.M. (2009). Age-associated increases in pulmonary artery systolic pressure in the general population. Circulation.

[37] Redfield M.M., Jacobsen S.J., Borlaug B.A., Rodeheffer R.J., Kass D.A. (2005). Age- and gender-related ventricular-vascular stiffening: A community-based study. Circulation.

[38] Kawaguchi M., Hay I., Fetics B., Kass D.A. (2003). Combined ventricular systolic and arterial stiffening in patients with heart failure and preserved ejection fraction: Implications for systolic and diastolic reserve limitations. Circulation.

[39] Curtis J.P., Sokol S.I., Wang Y., Rathore S.S., Ko D.T., Jadbabaie F., Portnay E.L., Marshalko S.J., Radford M.J., Krumholz H.M. (2003). The association of left ventricular ejection fraction, mortality, and cause of death in stable outpatients with heart failure. J. Am. Coll. Cardiol..

[40] Rosch S., Kresoja K.P., Besler C., Fengler K., Schöber A.R., von Roeder M., Lücke C., Gutberlet M., Klingel K., Thiele H. (2022). Characteristics of Heart Failure With Preserved Ejection Fraction Across the Range of Left Ventricular Ejection Fraction. Circulation.

[41] Toma M., Ezekowitz J.A., Bakal J.A., O’Connor C.M., Hernandez A.F., Sardar M.R., Zolty R., Massie B.M., Swedberg K., Armstrong P.W. (2014). The relationship between left ventricular ejection fraction and mortality in patients with acute heart failure: Insights from the ASCEND-HF Trial. Eur. J. Heart Fail..

[42] Ohte N., Kikuchi S., Iwahashi N., Kinugasa Y., Dohi K., Takase H., Masai K., Inoue K., Okumura T., Hachiya K. (2023). Unfavourable outcomes in patients with heart failure with higher preserved left ventricular ejection fraction. Eur. Heart J. Cardiovasc. Imaging.

[43] Sonaglioni A., Lonati C., Tescaro L., Nicolosi G.L., Proietti M., Lombardo M., Harari S. (2022). Prevalence and clinical outcome of main echocardiographic and hemodynamic heart failure phenotypes in a population of hospitalized patients 70 years old and older. Aging Clin. Exp. Res..

[44] Chantler P.D., Lakatta E.G. (2012). Arterial-ventricular coupling with aging and disease. Front. Physiol..

[45] Chen C.H., Nakayama M., Nevo E., Fetics B.J., Maughan W.L., Kass D.A. (1998). Coupled systolic-ventricular and vascular stiffening with age: Implications for pressure regulation and cardiac reserve in the elderly. J. Am. Coll. Cardiol..

[46] Lin Y., Fu S., Yao Y., Li Y., Zhao Y., Luo L. (2021). Heart failure with preserved ejection fraction based on aging and comorbidities. J. Transl. Med..

[47] Maurer M.S., King D.L., El-Khoury Rumbarger L., Packer M., Burkhoff D. (2005). Left heart failure with a normal ejection fraction: Identification of different pathophysiologic mechanisms. J. Card. Fail..

[48] Russo C., Jin Z., Palmieri V., Homma S., Rundek T., Elkind M.S., Sacco R.L., Di Tullio M.R. (2012). Arterial stiffness and wave reflection: Sex differences and relationship with left ventricular diastolic function. Hypertension.

[49] Bridges C.R., Edwards F.H., Peterson E.D., Coombs L.P., Ferguson T.B. (2003). Cardiac surgery in nonagenarians and centenarians. J. Am. Coll. Surg..

[50] Arsalan M., Szerlip M., Vemulapalli S., Holper E.M., Arnold S.V., Li Z., DiMaio M.J., Rumsfeld J.S., Brown D.L., Mack M.J. (2016). Should Transcatheter Aortic Valve Replacement Be Performed in Nonagenarians?: Insights From the STS/ACC TVT Registry. J. Am. Coll. Cardiol..

[51] Weinberg L., Walpole D., Lee D.K., D’Silva M., Chan J.W., Miles L.F., Carp B., Wells A., Ngun T.S., Seevanayagam S. (2022). Modern Cardiac Surgical Outcomes in Nonagenarians: A Multicentre Retrospective Observational Study. Front. Cardiovasc. Med..

[52] Fukuta H., Goto T., Wakami K., Ohte N. (2017). The effect of beta-blockers on mortality in heart failure with preserved ejection fraction: A meta-analysis of observational cohort and randomized controlled studies. Int. J. Cardiol..

[53] Mitchell A., Elmasry Y., van Poelgeest E., Welsh T.J. (2023). Anticoagulant use in older persons at risk for falls: Therapeutic dilemmas-a clinical review. Eur. Geriatr. Med..

[54] Lefebvre M.C., St-Onge M., Glazer-Cavanagh M., Bell L., Kha Nguyen J.N., Viet-Quoc Nguyen P., Tannenbaum C. (2016). The Effect of Bleeding Risk and Frailty Status on Anticoagulation Patterns in Octogenarians with Atrial Fibrillation: The FRAIL-AF Study. Can. J. Cardiol..

[55] Ferrazzini E., Méan M., Stalder O., Limacher A., Rodondi N., Aujesky D. (2023). Incidence and clinical impact of bleeding events in older patients with acute venous thromboembolism. Blood Adv..

[56] Potter E., Marwick T.H. (2018). Assessment of Left Ventricular Function by Echocardiography: The Case for Routinely Adding Global Longitudinal Strain to Ejection Fraction. JACC Cardiovasc. Imaging.

